# Thyroid Diagnosis from SPECT Images Using Convolutional Neural Network with Optimization

**DOI:** 10.1155/2019/6212759

**Published:** 2019-01-15

**Authors:** Liyong Ma, Chengkuan Ma, Yuejun Liu, Xuguang Wang

**Affiliations:** ^1^School of Information Science and Engineering, Harbin Institute of Technology, Weihai 264209, China; ^2^School of Automation, Harbin University of Science and Technology, Harbin 150080, China; ^3^Nuclear Medicine Department, Heilongjiang Provincial Hospital, Harbin 150030, China

## Abstract

Thyroid disease has now become the second largest disease in the endocrine field; SPECT imaging is particularly important for the clinical diagnosis of thyroid diseases. However, there is little research on the application of SPECT images in the computer-aided diagnosis of thyroid diseases based on machine learning methods. A convolutional neural network with optimization-based computer-aided diagnosis of thyroid diseases using SPECT images is developed. Three categories of diseases are considered, and they are Graves' disease, Hashimoto disease, and subacute thyroiditis. A modified DenseNet architecture of convolutional neural network is employed, and the training method is improved. The architecture is modified by adding the trainable weight parameters to each skip connection in DenseNet. And the training method is improved by optimizing the learning rate with flower pollination algorithm for network training. Experimental results demonstrate that the proposed method of convolutional neural network is efficient for the diagnosis of thyroid diseases with SPECT images, and it has superior performance than other CNN methods.

## 1. Introduction

The thyroid gland is one of the important organs of the human body. It produces the thyroid hormone which is vital to control the body's metabolism. Thyroxine and triiodothyronine are two active thyroid hormones that have important effects on protein production, body temperature regulation, energy production, and energy regulation of the human body. Therefore, if the thyroid gland is diseased, the metabolism and regulation of the human body will lose the necessary control and that may be life threatening in severe cases.

Thyroid disease has now become the second largest disease in the endocrine field [1], which can lead to death when the disease is severe. The total number of patients with thyroid disorders worldwide is more than 300 million, of whom the number of females is about 6∼10 times of that of male patients, and the number of females over 40 years is about 10%∼20%. In China, there are more than 40 million people with primary hypothyroidism and more than 10 million people with primary hyperthyroidism. The treatment rate for hyperthyroidism in China is less than 5%.

In practical clinical practice, many approaches can be used to diagnose thyroid diseases, such as clinical evaluation, blood examination, thyroid hormone (TSH) detection, imaging examination, and tissue biopsy. The comprehensive application of various detection methods has been very common in clinical diagnosis, such as the combined use of TSH detection data and blood examination data.

Computer-aided diagnosis (CAD) systems are increasingly being used in clinical diagnostics. On the one hand, these CAD systems can reduce the drudgery of doctors. On the other hand, they can avoid some mistakes that may be made in the diagnostic process. More and more CAD systems are being applied in practice to improve the accuracy of diagnosing various diseases. A lot of research studies on CAD of thyroid diseases have been carried out at present [2–10].

Imaging technology is very important for the diagnosis of thyroid diseases, so there have been a lot of research studies on CAD for imaging technology. Common medical images used to diagnose thyroid diseases include ultrasound, CT, SPECT, etc. Ultrasound imaging has the advantages of good real time, convenient operation, and low cost, so it is widely used in the clinical diagnosis of thyroid diseases. The CAD of thyroid disease based on ultrasonography was developed earlier, and the typical example was a benign or malignant diagnosis of thyroid nodules based on ultrasound [8–10]. Compared with the ultrasonic image, the CT image has a clearer image detail display, but it also brings ionizing radiation. CT images also caught attention in CAD studies, such as image segmentation and volume estimation of the thyroid gland [11]. Although as an important imaging modality, SPECT plays an irreplaceable role in the diagnosis of thyroid diseases, there is no research on the CAD of thyroid diseases based on SPECT.

Unlike ultrasound imaging and X-ray imaging of CT, SPECT imaging uses a gamma-ray camera to collect image data, and it is a nuclear medicine imaging method. The SPECT imaging system consists of one or more gamma cameras mounted on the platform that allows these cameras to accurately rotate around the patient when collecting the images. Patients ingest radioisotope drugs with appropriate half lives. Due to radioactive decay, drugs emit gamma photons when they reach the desired imaging location. The main feature of SPECT imaging is that the resulting images are 3D tomographic images, which can provide various cross section information. SPECT can show the changes of blood flow, function, and metabolism of organs or lesions, which is beneficial to the early diagnosis and diagnosis of the disease. SPECT imaging is particularly important for the clinical diagnosis of thyroid diseases. At present, when other imaging or examination methods cannot provide a reliable diagnosis, it is necessary to use SPECT imaging to make a final diagnosis of thyroid diseases. Although SPECT is extremely important for the diagnosis of thyroid diseases, there is no study of thyroid disease CAD using SPECT image currently.

This paper studies thyroid disease CAD using SPECT image. In this paper, the machine learning method of deep learning is adopted to diagnose thyroid diseases using SPECT images. From the perspective of machine learning methods, the use of SPECT images for thyroid disease diagnosis is to link the characteristics of SPECT images with the diagnosis of thyroid diseases, and the classification problem of SPECT is to classify thyroid SPECT images into specific diseases according to characteristics. Therefore, for machine learning methods, the use of SPECT images for disease diagnosis is to solve the classification problem of SPECT images. The DenseNet network is an important deep learning network architecture that has emerged in recent years, and it has performed well in many practical applications. This paper uses DenseNet network to establish the diagnosis model of thyroid disease based on SPECT image. On the basis of the traditional DenseNet network architecture, both the architecture and the training method are improved in this paper, which greatly improves the diagnosis effect of thyroid disease.

The main contributions of the paper include the following: first, the paper introduces the deep learning method into the diagnosis of thyroid disease based on SPECT images. Second, the paper has improved the existing deep learning network DenseNet from both the network architecture and the training method, which makes the diagnostic effect of the deep learning method greatly improved compared with other deep learning methods.

This paper is organized as follows.  Section 2 reviews the related works.  Section 3 presents the proposed solution based on deep learning method for thyroid disease diagnosis using SPECT images.  Section 4 presents experimental results and discussions.  Section 5 concludes the paper.

## 2. Related Work

In the CAD study of thyroid disease, a large amount of literature focuses on the diagnostic research employing TSH data due to the open dataset of TSH in the UCI machine learning repository. In this dataset, thyroid diagnosis is considered as a classification problem with three classes of normal, hyperthyroidism, and hypothyroidism. If we consider the diagnosis of thyroid disease as a classification problem, we can introduce a powerful machine learning technology to discover the complex relationship of biomedicine to build CAD system. With the development of machine learning technology, the recognition accuracy of this problem has been improved gradually. The diagnosis accuracy was 78.14% when employing probabilistic neural network method in 1997 [2]. The accuracy achieved 88.3% when multilayer perception was used in 2004 [3]. The accuracy rate of wavelet-based support vector machine (SVM) recognition was 91.86% in 2011 [4], and the SVM method with particle swarm optimization achieved the accuracy of 97.49% in 2012 [5]. And extreme learning machine method (ELM) has achieved the accuracy rate of 97.73% [6].

Deep learning is a branch of machine learning technology in the field of artificial intelligence [12, 13]. Traditional machine learning techniques, including neural networks, SVM, ELM, etc., do not work well with direct processing of raw data. However, deep learning technology can automatically discover the feature expression and classification methods which need to be detected and classified by calculation, thus greatly improving the effectiveness of machine learning. Deep learning is such a feature learning method, which transforms the original data into a higher level and more abstract expression through some simple nonlinear models. Deep learning uses a combination of enough transformations so that very complex functions can also be learned.

The deep learning architecture uses a multilayer stack of simple modules, and most of which are aimed at learning, as well as some mappings for calculating nonlinear input and output. Each module in the stack converts its input to increase the selectivity and immutability of the expression. Convolution neural network (CNN) is a typical depth learning model, which consists of a series of modules. The initial modules are composed of a convolution layer, a pool layer, and the elements of the convolution layer. The function of convolution layer is to detect the local connection of the upper layer feature, and the function of the pool layer is to combine the similar features in semantics. The important feature of deep learning is that the features of each layer are not designed artificially but are learned from the data using the common learning process. As one of the most important structures of deep learning, CNN is no exception. This ability to automatically discover features makes CNN achieve better results than traditional methods in many applications such as disease diagnosis based on medical image classification and lesion area segmentation. CNN is also used in a variety of medical imaging [14–16], such as ultrasound, CT, MRI, medical optics images, photoacoustic imaging, and so on.

In recent years, methods based on deep learning have also been used for the recognition and processing of thyroid images. Because of the high incidence of thyroid nodules, the use of ultrasound images to detect thyroid nodules is currently more studied using CNN methods [10,17–19]. These studies use the usual CNN model or a combination of two CNN model. The CNN method is also used to detect thyroid cancer using ultrasound images. The identification of thyroid papillary cancer and thyroid papillary carcinoma using ultrasound images was reported [20, 21]. Simple CNN models have also been used in these studies.

At present, the CNN method has developed many complex and effective models, which have also been gradually applied to medical diagnosis. DenseNet [22] is an important CNN architecture, and it has been widely used for disease diagnosis. For example, an efficient cardiac disease classification employing DenseNet is reported recently in [23]. ResNet is another efficient CNN architecture [24], and it is used to classify clinical 12 skin diseases in recent times [25]. Inception model is also reported for MRI-based classification of migraine in [26]. VGG architecture of CNN is employed in two-phase multimodel automatic brain tumour diagnosis system [27] and lung nodule classification between benign nodule and lung cancer [28]. And a VGG variant architecture is employed to detect breast cancer using symmetry information [29]. GoogleNet is a convolutional neural network with a standard stacked convolutional layer with one or more fully connected layers. It has many successful applications in image-based medical diagnosis, such as identifying the stage of diabetic retinopathy [30], automated classification of pulmonary tuberculosis in chest radiography [31], the classification of breast lesions in ultrasound images [32], and the like. In particular, Chi et al. studied thyroid nodule classification in ultrasound images based on GoogleNet [9]. The stacked denoising autoencoders (SDAE) model increases the robustness of the model by introducing noise into the input layer and is also used in medical diagnostics such as lung cancer diagnosis and brain lesion detection [33–35]. Studies have shown that the applications of these models help CNN to better discover the characteristics of different image types, and thus obtain better classification results. In the research of this paper, we improve the network architecture and the training method based on DenseNet model. The experimental results show that this method has higher accuracy and better performance than other models in thyroid disease classification using SPECT image.

## 3. Materials and Methods

Different from the modality of ultrasound and CT that are often used to determine existing nodules and the nature of nodules, SPECT is more often used to determine the functional state of the thyroid gland in clinical practice. Classification of thyroid diseases by pathology is very complex. However, in actual clinical diagnosis and treatment, thyroid disorders are usually divided into three categories, which are Grave's disease, Hashimoto disease, and subacute thyroiditis. To be consistent with the actual clinical needs, the diagnosis based on SPECT images in this research is also divided into four categories, which are Grave's disease, Hashimoto disease, subacute thyroiditis, and normality.

**Figure 1:**
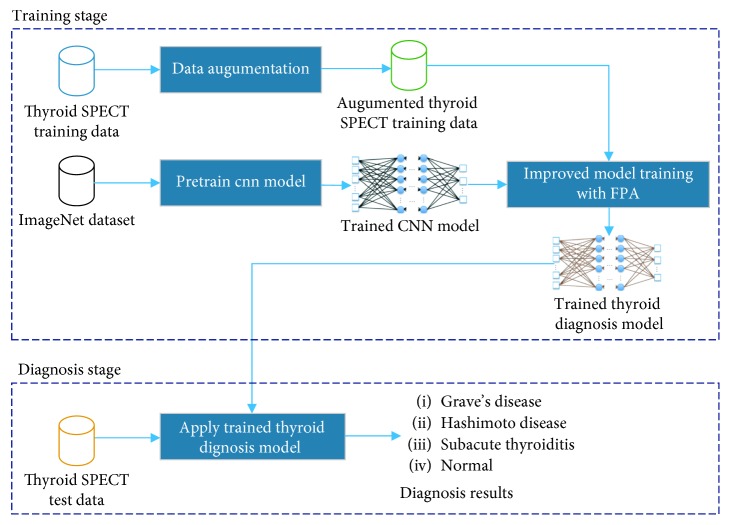
The proposed thyroid SPECT diagnosis method.

The proposed thyroid SPECT diagnosis method is illustrated in Figure 1.

Like other machine learning methods, the deep learning-based approach presented in this paper is divided into two stages: training stage and diagnosis stage. In the training phase, we used data augumentation, transfer learning, improved network architecture, and optimization-based training methods to train the CNN model. We will discuss these techniques in detail below. When the training is completed, this trained model can be used for SPECT-based thyroid disease diagnosis. When a SPECT image is input, the model can output the diagnosis result, which is Grave's disease, Hashimoto disease, subacute thyroiditis, or normality.

### 3.1. Dataset and Data Augumentation

**Figure 2:**
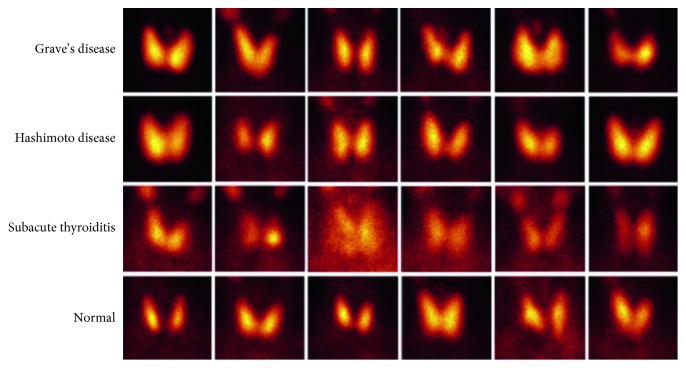
Image samples in the dataset.

A SPECT image dataset was established for the diagnosis of thyroid diseases. The images were collected with Siemens SPECT ECAM in Heilongjiang Provincial Hospital. The sources of these images were outpatient or inpatient. These SPECT images were also labelled with the true thyroid diagnosis results. The labelled diagnostic conclusions were confirmed by the medical history and the auxiliary examination, and many of these conclusions are confirmed by the cure after diagnosis and treatment. Some images in the dataset are illustrated in Figure 2. The dataset had 780 samples of Grave's disease, 438 samples of Hashimoto disease, 810 samples of subacute thyroiditis, and 860 samples of normality.

Due to the fact that the dataset was not large enough, mixup [36, 37] was employed to augment the dataset. Mixup method can generate new samples by linear interpolation of given samples and their labels. Many studies have revealed that the mixup training method has better generalization ability than the traditional empirical risk minimization method [36, 37]. And mixup is used in this paper to generate more samples. In mixup, two images were selected each time and were linearly interpolated to generate a new virtual sample as follows:

(1) $$\begin{array}{c} X^{\prime }=\alpha X_{i}+\left(1-\alpha \right)X_{j},\\ Y^{\prime }=\alpha Y_{i}+\left(1-\alpha \right)Y_{j}, \end{array}$$

where *X*_*i*_ is an original image which is randomly selected from one SPECT category to be augmented, *X*_*j*_ is an original image which is randomly selected from all the SPECT image dataset, and *X*_*j*_ is different from *X*_*i*_. *Y*_*i*_ and *Y*_*j*_ are one-hot encoding vectors to represent the corresponding category of *X*_*i*_ and *X*_*j*_ respectively, and *α* and (1 − *α*) are the linear factors of *X*_*i*_, *X*_*j*_ and *Y*_*i*_, *Y*_*j*_, respectively, 0.5 ≤ *α* ≤ 1; they determine the proportion of the original two real samples when generating a new fake sample.

We have increased the samples of the dataset. When we generate a new sample, *α* is randomly selected from a uniform distribution of [0.5, 1]. And each kind of sample number reaches to 2000. Every category is randomly divided into 2 groups, among them 1400 samples for training and 600 samples for test.

### 3.2. Transfer Learning

As obtaining large dataset with comprehensively annotation in the medical imaging domain is a challenge, transfer learning is often employed to solve the problem of lacking data. Transfer learning fine-tunes CNN models pretrained from natural image dataset to medical image tasks. The effectiveness of transfer learning in medical image classification and detection tasks has been demonstrated in many studies and applications [17, 28, 38]. When a small dataset is trained directly with deep learning networks, it can easily lead to overfitting. The transfer learning is able to improve the initial ability of extracting features to alleviate the risk of overfitting. We transferred a set of pretrained weight from ImageNet to our proposed network. After the transferring of network weights, we can use the SPECT image dataset to fine-tune our proposed network. Similar to other medical image classification and detection tasks, we used batch normalization to normalize the batch to prevent the gradient from vanishing or exploding in the fine-tune process. In order to prevent overfitting, we adopt dropout strategy in the training process where each backpropagation updates a part of the network with a certain probability. We also take early stopping strategy to stop training when the network performance on the validation set no longer increases.

### 3.3. Network Architecture

DenseNet is a recently proposed network architecture that has been studied and applied to provide more effective classification accuracy than previous networks. The advantage of DenseNet is that it alleviates the problem of gradient vanishment. The gradient vanishment problem is due to the use of backward propagation in deep learning networks to modify the parameters by calculating gradients to reduce the classification error. But with the deepening of the network depth, the gradient will gradually disappear in backward propagation. Under the premise of guaranteeing the maximum information transmission between the layers in the middle, DenseNet can alleviate the problem of gradient vanishment by directly connecting all the layers. Meanwhile, DenseNet enhances the delivery of the feature and makes more efficient use of the feature. Finally, DenseNet is somewhat less parametric, making it easier to be trained. Therefore, in the research of this paper, DenseNet is also used to classify thyroid diseases based on SPECT images.

In DenseNet, the output of the *l*-th layer is

(2) $$\begin{array}{c} y_{l}=F_{l}\left(\left[x_{0},x_{1},\ldots ,x_{l-1}\right],\left\{W_{l}\right\}\right), \end{array}$$

where *F*_*l*_ is a nonlinear transformation function, *W*_*l*_ is its parameters in the *l*-th block, *y*_*l*_ is the output of the *l*-th block, *x*_*l*_ is the input of the *l*-th block, and [*x*_0_, *x*_1_,…, *x*_*l*−1_] refers to the concatenation of the features produced in layers 0,1,…, *l* − 1.

**Figure 3:**
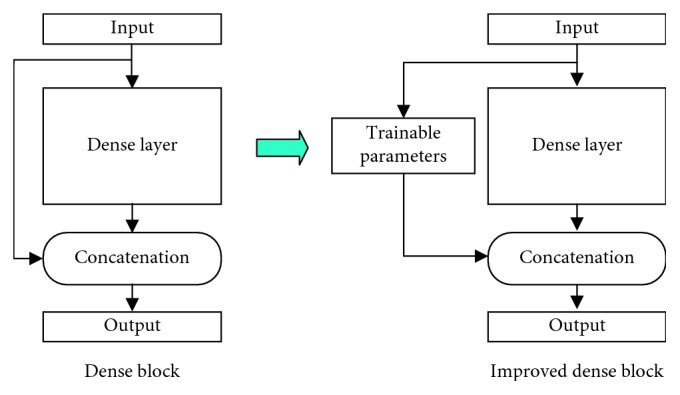
The improved dense block replaces all the features with trainable parameters for concatenation.

A modified network architecture is developed in this paper. In DenseNet, features of the previous layers are concatenated with the same weight in every cross layer, but not all these previous features are useful. A modified architecture is proposed by adding the trainable weight parameters to each skip connection as shown in Figure 3. And the output of the *l*-th layer in this modified structure is modified as

(3) $$\begin{array}{c} y_{l}=F_{l}\left(\left[x_{0}\cdot k_{l,0},x_{1}\cdot k_{l,1},\ldots ,x_{l-1}\cdot k_{l,l-1}\right],\left\{W_{l}\right\}\right), \end{array}$$

where *k*_*l*,0_, *k*_*l*,1_,…, *k*_*l*,*l*−1_ refers to the parameters which determinate weights of *x*_0_, *x*_1_,…, *x*_*l*−1_ when they concatenate to the *l*-th layer.

**Figure 4:**
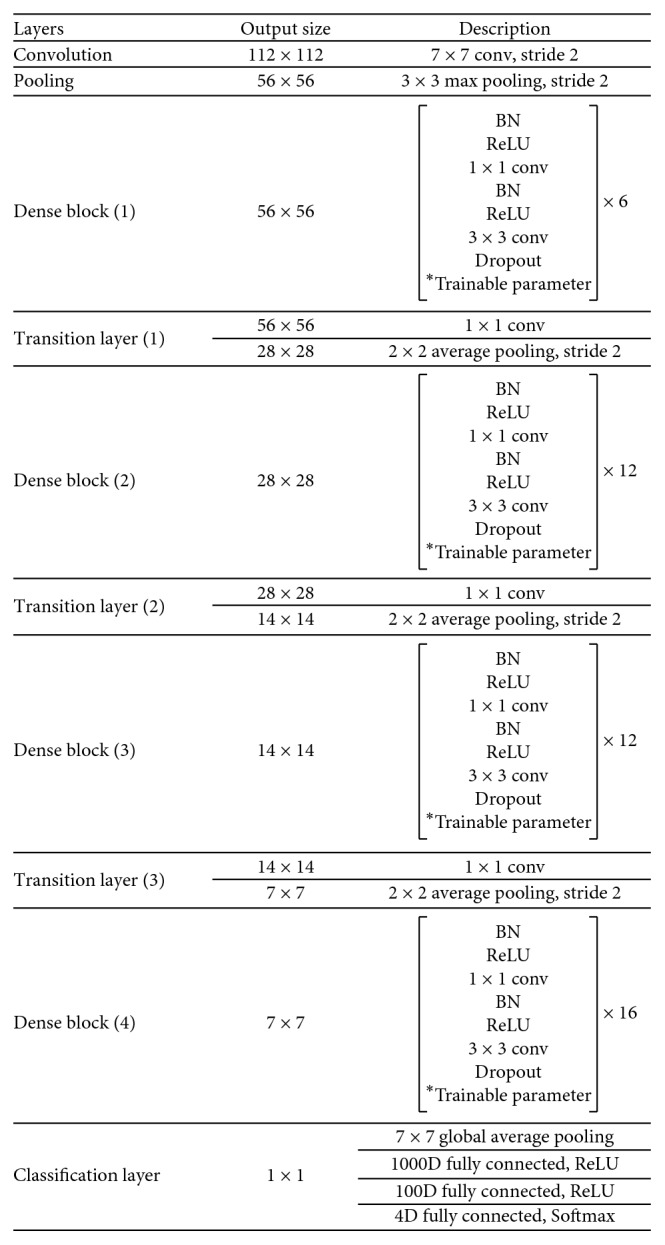
The CNN architecture of the proposed thyroid SPECT diagnosis method.

**Figure 5:**
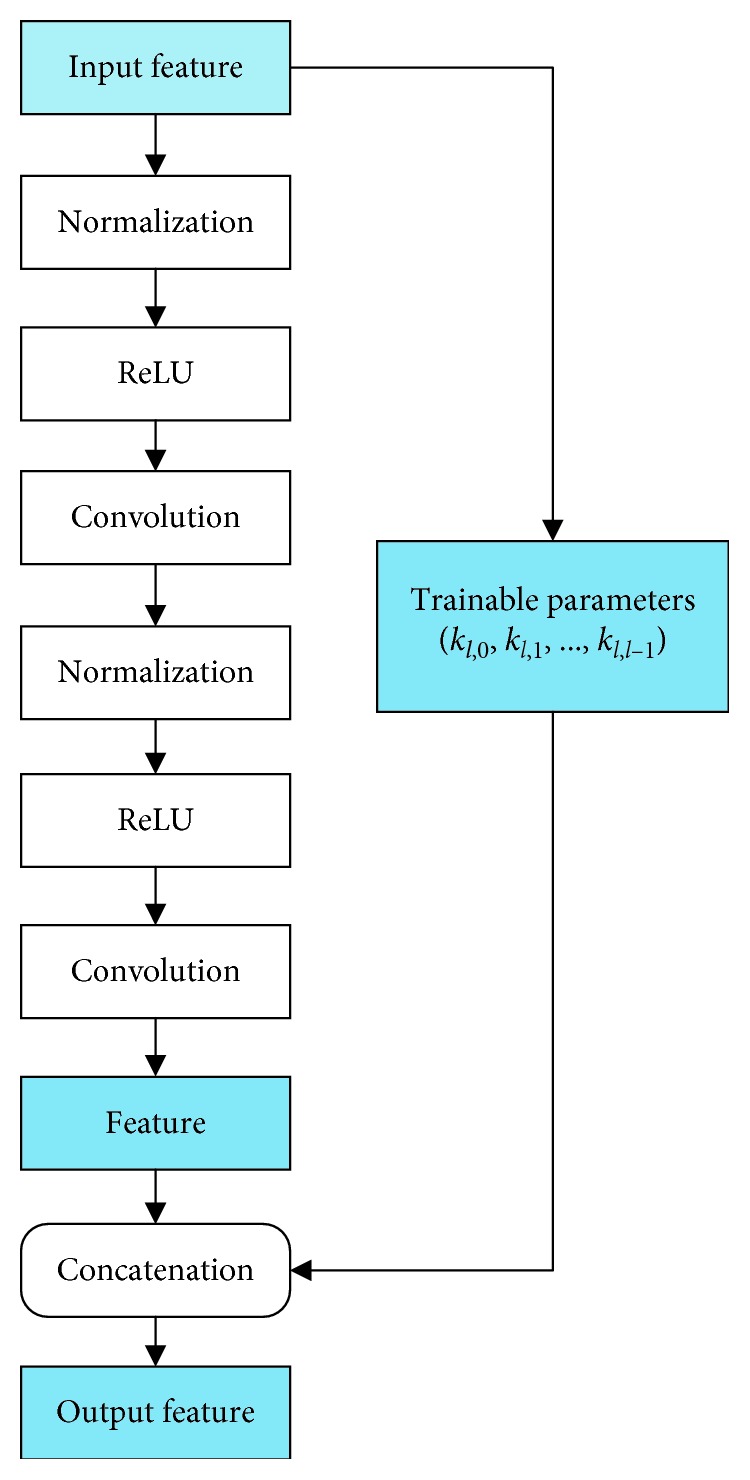
The improved dense block architecture of the proposed thyroid SPECT diagnosis method.

The detailed network architecture is listed in Figure 4. And the dense block in the architecture is the improved dense block, and the architecture of the improved dense block is shown in Figure 5.

In the proposed architecture, the network is more easy to learn the weights parameters during the training period. During the process of backward propagation, the parameters of weight represent the degree of importance of the corresponding feature map for the image classification. Therefore, greater weight means the corresponding feature map plays a vital role in the classification task, that is to say it contains more useful information. Otherwise, the weight is small. Due to the fact that the weights of the corresponding feature map in each layer are no longer fixed, the network obtains greater flexibility with the ability to enhance effective features. Meanwhile, the pooling layer is replaced with dilated convolution layer in the network architecture to preserve the important features as much as possible.

### 3.4. Learning Rate Optimized with Flower Pollination Algorithm

Learning rate is one of the most important parameters of CNN, and the quality of learning rate selection will largely determine the speed and quality of network training. The random gradient descent algorithm is used in CNN, and the training set is divided into several minibatches. For each minibatch, the following processing procedure is repeated. The minibatch is used as the input *x* of the network, and the output *y* is computed, that is,

(4) $$\begin{array}{c} y=f\left(x,w\right), \end{array}$$

where *w* is the network parameter. Then, we compare the output *y* with the label $\overset{⌢}{y}$ representing the true value with the loss function *L* to get the loss *C*. Finally, by calculating the gradient value ∇*w*=∂*C*/∂*w* of the network parameters, we use this gradient to update *w* as

(5) $$\begin{array}{c} w\;⟵\;w-l\cdot \nabla w, \end{array}$$

where *l* is learning rate.

However, there are two problems with the above method. First, after updating the parameter *w* of the current minibatch, the processing and parameter updating of the next minibatch data begins directly, while the effect of the parameter updating of the minibatch is not validated. This means that it is not known whether the loss *C* of the current minibatch is reduced. Second, since the learning rate is often selected manually based on experience, it is likely that it does not effectively reduce the computational loss of the current minibatch.

Therefore, this paper proposes an improved method to solve the two problems mentioned above. Before updating with equation (5), the learning rate *l* in equation (5) is optimized to get the best learning rate parameter, and then equation (5) is applied to complete the update. By applying this optimization strategy, we can ensure that each updated *w* can reduce the loss value of the current minibatch, which is a better classification result, so as to ensure a better learning effect.

In this paper, the flower pollination algorithm (FPA) algorithm is used to find the optimal parameter of learning rate *l* in equation (5). FPA algorithm is a kind of optimization algorithm which simulates the pollination process of flowers in nature, which has been used to solve multiobjective optimization problem and has achieved good results [39–41]. The algorithm has the advantages of less parameters and easier implementation, and it has strong global optimization ability.

Pollen pollination in nature includes self-pollination and cross-pollination, which is simulated by FPA algorithm. In the FPA algorithm, it is assumed that each flower is a solution of the optimization problem solved; each flower chooses the cross-pollination operation with the probability *P*_C_ to reproduce or chooses the self-pollination operation with the probability 1 − *P*_C_ to reproduce. Cross-pollination operation draws on the method of cross-pollination in the nature of bee and butterflies in different flowers with levy distribution in the global pollination of flowers. Self-pollination operation simulates the method of near-distance local pollination between the same flower in nature. The main optimization process of learning rate parameters based on FPA in this paper is summarized as follows.

***Step 1*.**

*Parameter Initialization*. Determine the number of iterations *N*, the number of pollen *m*, and the probability of *P*_C_; the range of learning rate *l* is in [*L*_min_, *L*_max_]. And *l* is regarded as the pollen, which obeys the standard distribution on the interval of [*L*_min_, *L*_max_].

***Step 2*.**

Probability *P* is randomly generated, and if *P* ≤ *P*_C_, the cross-pollination operation is performed; otherwise the self-pollination operation is performed. In cross-pollination operation, the following update of the current learning rate *l*_*i*_ is performed

(6) $$\begin{array}{c} l_{i}\;⟵\;l_{i}+\gamma \cdot L\cdot \left(l_{\text{best}}-l_{i}\right), \end{array}$$

where *γ* is the scaling factor, and in this paper it is set to 0.1. *L* is a random number which subjects to the Levy distribution with the exponential parameter of 1.5. *l* is the current learning rate, and *l*_best_ is the optimal solution in the global. In self-pollination operation, the following update of the current learning rate *l*_*i*_ is performed:

(7) $$\begin{array}{c} l_{i}\;⟵\;l_{i}+\varepsilon \cdot \left(l_{1}-l_{2}\right), \end{array}$$

where *ε* is a random real number subject to uniform distribution between [0,1] and *l*_1_ and *l*_2_ are two random pollens, which denote learning rates in our application.

**Figure 6:**
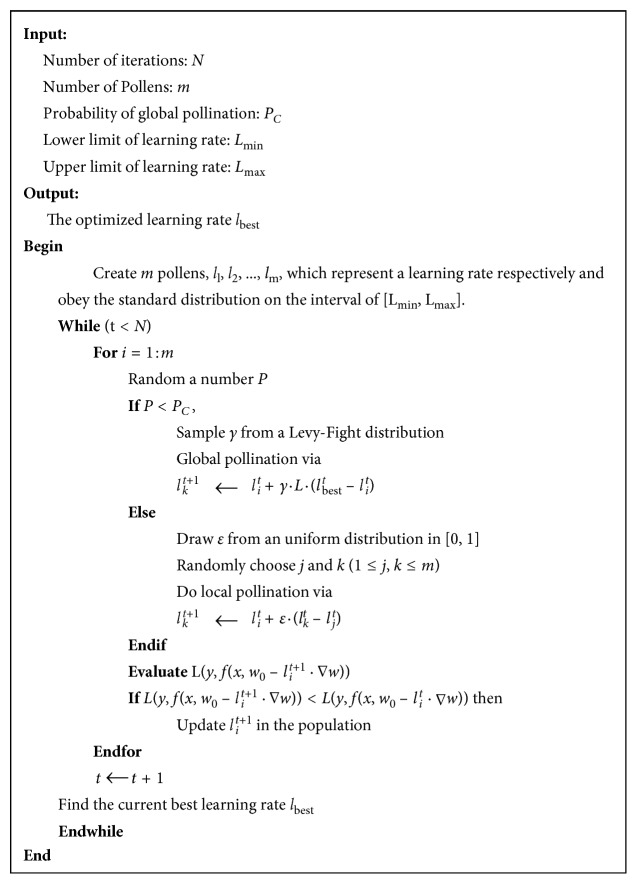
Pseudocode of flower pollination algorithm used for updating learning rate.

The pseudocode of flower pollination algorithm used for updating learning rate is shown in Figure 6. After applying the above mentioned FPA algorithm to optimize learning rate parameter in CNN training, we can obtain a well-trained CNN with good performance for classification of thyroid disease with SPECT images.

## 4. Results and Discussion

The experiments are performed using the deep learning framework PyTorch on a workstation equipped with two NVIDIA Geforce 1080Ti GPUs and an Intel Xeon E5-2620 CPU. Transfer learning is used with DenseNet121 and parameters that were pretrained by ImageNet and fine-tuned with our SPECT image dataset. Each minibatch contains 5 images, and each image size is 255 × 255. The loss function is set as cross entropy loss. Every experiment is preformed 10 times, and results are averaged.

Other CNN methods, including DenseNet121 [22], ResNet101 [23], InceptionV3 [24], VGG19 [25, 26], Modified VGG (MVGG) [27], GoogleNet [9,28–30], and SDAE [31–33], have also been implemented for comparison with the methods proposed in this paper.

### 4.1. Classification Performance Evaluation

In order to evaluate our proposed method, the classification recall, precision, accuracy, specificity, and F1 score are used as the indicators to evaluate the performances of different methods.

Depending on whether the classification results of the CNN are correct and whether the sample is positive, true positive (TP), true negative (TN), false positive (FP), and false negative (FN) for each class can be determined. Recall, precision, accuracy, and F1 score are defined as follows:

(8) $$\begin{array}{c} \text{recall}=\frac{\text{TP}}{\text{TP}+\text{FN}},\\ \text{precision}=\frac{\text{TP}}{\text{TP}+\text{FP}},\\ \text{accuracy}=\frac{\text{TP}+\text{TN}}{\text{TP}+\text{FN}+\text{FP}+\text{TN}},\\ \text{specificity}=\frac{\text{TN}}{\text{FP}+\text{TN}},\\ F1\text{ score}=\frac{2\text{TP}}{2\text{TP}+\text{FP}+\text{FN}}. \end{array}$$

Recall is the ratio of the number of samples correctly predicted for the class to the total number of samples for the class, and it is also called sensitivity or hit rate. Precision refers to the ratio of the number of category samples correctly predicted to the total number of samples all predicted for that category. Accuracy is the ratio of correctly predicted observations. Specificity is the ratio of correctly predicted negative samples to the total negative samples. F1 score is the harmonic mean of precision and sensitivity of the classification. The larger these performance values are, the better the performance of a method is.

To compare the classification accuracy, the classification recall, precision, accuracy, specificity, and F1 score indicators are listed in Tables 1–4. Our proposed method obtains the best performance than other methods. This shows that our improvements to the network architecture and learning method are effective.

### 4.2. Average Precision with Different Iteration Numbers

**Figure 7:**
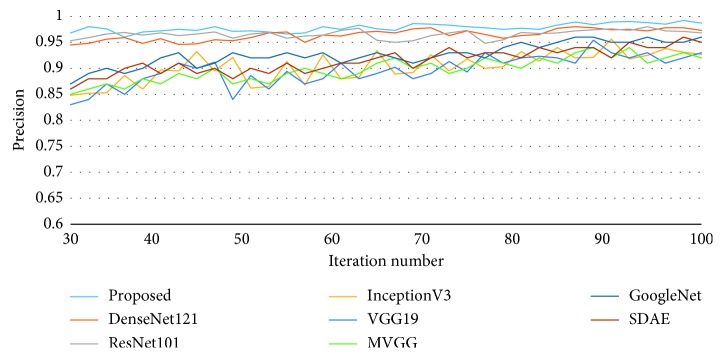
Average precision curve with different iteration numbers.

Average precision with different iteration numbers is also compared and illustrated in Figure 7. Our proposed method obtains the best average precision when the iteration number is greater than 56, so the overall performance of our proposed method is superior to other methods.

### 4.3. Confusion Matrix

**Figure 8:**
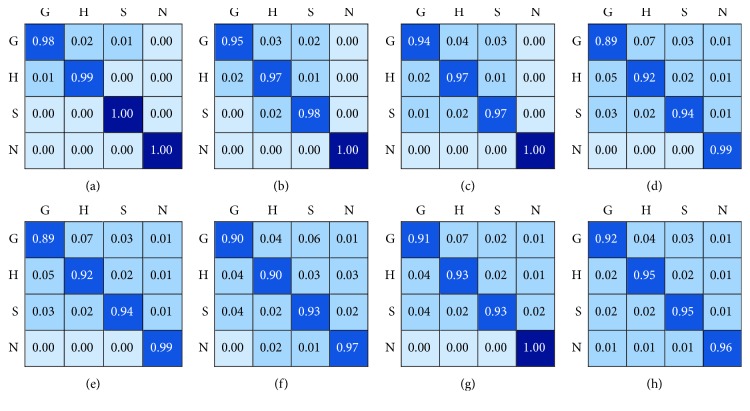
Confusion matrix comparison.

The confusion matrix is also employed in this work to evaluate the classification performance. Each column represents the actual category that images are classified to in confusion matrix. And the total number of each column represents the number of images actually classified as the category. The confusion matrix of different methods in this work is illustrated in Figure 8. And our proposed method has the least classification error.

## 5. Conclusions

An efficient method of convolutional neural network for the diagnosis of thyroid diseases using SPECT images is proposed. The proposed method employs modified DenseNet architecture and improved training method. Experimental results demonstrate that the proposed method had superior performances than other methods of convolutional neural network. This study shows that it is feasible to apply convolutional neural network to the diagnosis of thyroid diseases based on SPECT images, and the method presented in this paper is very promising.

And there is still much work to be done before CNN-based method can be applied in practice, including the establishment and testing of larger datasets, the comparison and evaluation of diagnoses by professional physicians, and the study of more effective models of deep learning. In addition, due to insufficient data, more detailed classification and diagnosis of thyroid diseases has not been carried out, which will be a very important part of our future research.

## Figures and Tables

**Table 1:** Grave's disease class performance comparison of different methods (percent).

Network	DenseNet121	ResNet101	InceptionV3	VGG19	MVGG	GoogleNet	SDAE	Proposed
Recall	95.17	93.83	88.50	89.00	89.50	90.83	92.33	**97.50**
Precision	98.11	98.57	90.15	91.44	91.95	91.91	94.22	**98.82**
Accuracy	98.33	97.63	94.71	95.17	95.42	95.71	96.67	**99.08**
Specificity	99.39	98.89	96.78	97.22	97.39	97.33	98.11	**99.61**
F1 score	96.62	95.18	89.32	90.20	90.71	91.37	93.27	**98.15**

**Table 2:** Hashimoto disease class performance comparison of different methods (percent).

Network	DenseNet121	ResNet101	InceptionV3	VGG19	MVGG	GoogleNet	SDAE	Proposed
Recall	97.17	96.50	92.33	91.67	90.33	93.17	94.50	**98.50**
Precision	95.57	94.92	90.67	91.21	91.71	91.49	93.41	**98.50**
Accuracy	98.17	97.83	95.71	95.71	95.54	96.13	96.96	**99.25**
Specificity	98.50	98.28	96.83	97.06	97.28	97.11	97.78	**99.50**
F1 score	96.37	95.70	91.49	91.44	91.02	92.32	93.95	**98.50**

**Table 3:** Subacute disease class performance comparison of different methods (percent).

Network	DenseNet121	ResNet101	InceptionV3	VGG19	MVGG	GoogleNet	SDAE	Proposed
Recall	98.17	97.33	89.17	94.00	92.50	92.83	95.33	**100.00**
Precision	96.88	96.21	93.04	94.31	90.54	96.20	93.77	**98.68**
Accuracy	98.75	98.38	95.63	97.08	95.71	97.29	97.25	**99.67**
Specificity	98.94	98.72	97.78	98.11	96.78	98.78	97.89	**99.56**
F1 score	97.52	96.77	91.06	94.16	91.51	94.49	94.55	**99.34**

**Table 4:** Normal class performance comparison of different methods (percent).

Network	DenseNet121	ResNet101	InceptionV3	VGG19	MVGG	GoogleNet	SDAE	Proposed
Recall	100.00	100.00	99.50	99.17	96.83	100.00	96.33	**100.00**
Precision	100.00	100.00	95.52	96.75	94.93	97.24	97.14	**100.00**
Accuracy	100.00	100.00	98.71	98.96	97.92	99.29	98.38	**100.00**
Specificity	100.00	100.00	98.44	98.89	98.28	99.06	99.06	**100.00**
F1 score	100.00	100.00	97.47	97.94	95.87	98.60	96.74	**100.00**

## Data Availability

The data used to support the findings of this study are available from the corresponding author upon request.
